# European citizens’ perspectives on direct-to-consumer genetic testing: an updated systematic review

**DOI:** 10.1093/eurpub/ckz246

**Published:** 2020-05-03

**Authors:** Ilda Hoxhaj, Jovana Stojanovic, Stefania Boccia

**Affiliations:** Sezione di Igiene, Istituto di Sanità Pubblica, Università Cattolica del Sacro Cuore, Roma, Italia; Sezione di Igiene, Istituto di Sanità Pubblica, Università Cattolica del Sacro Cuore, Roma, Italia; Department of Health, Kinesiology, and Applied Physiology, Concordia University, Montreal, Canada; Montreal Behavioural Medicine Centre, CIUSSS du Nord-de-l’Île-de-Montréal, Montréal, Canada; Sezione di Igiene, Istituto di Sanità Pubblica, Università Cattolica del Sacro Cuore, Roma, Italia; Department of Woman and Child Health and Public Health—Public Health Area, Fondazione Policlinico Universitario A. Gemelli IRCCS, Roma, Italia

## Abstract

**Background:**

Direct-to-consumer genetic tests (DTC-GTs) are genetic tests for a medical or non-medical trait that are sold directly to the public, usually ordered without the engagement of a healthcare professional. Our aim was to explore the knowledge, attitudes and behaviors toward DTC-GTs among European citizens.

**Methods:**

We updated the most recent systematic review on citizens’ perspectives toward DTC-GTs. Relevant English language studies were searched on PubMed, ISI Web of Science, Scopus, Embase and Google Scholar from October 2014 to April 2019. We extended our search on Scopus without publication date restriction, since it was not included in the former review. Eligible studies were conducted in European countries and reported original data. The quality of the studies was evaluated using a checklist developed by Kmet et al.

**Results:**

We included six studies conducted in European countries between 2015 and 2018. The studies were performed among general population in the Netherlands, students in Italy and Greece, laypeople in Germany and older adults in Switzerland. The level of awareness, in overall low, differed by country and population group. Most of the participants were interested in undergoing a DTC-GT, mainly for knowing the risk predisposition to a common disease. Concerns were raised about tests’ validity and utility and data privacy.

**Conclusions:**

Our review shows that European citizens, overall, have a low level of knowledge on DTC-GTs and a high interest in their purchase. This understanding might contribute to the development of educational programs in order to the increase of general public capabilities to make appropriate health decisions.

## Introduction

Rapid advancements in genomic knowledge have contributed to the development of new methods for predicting and preventing diseases.^1^ Over the years, the widespread access to internet has enabled an enormous number of companies to advertise genetic tests (GTs).^2^ Direct-to-consumer genetic tests (DTC-GTs) are tests ordered directly by the consumer, without the involvement of a healthcare professional.^3^ DTC-GTs provide a variety of information to the consumer, from ancestral connections and lifestyle to personal susceptibility to certain common diseases, such as diabetes, cardiovascular diseases (CVDs) or cancer.^4^^,^^5^ These tests usually analyze common DNA variants, which account for only a fraction of the heritable component of multifactorial diseases including cancer.^6^^,^^7^ Many concerns have been raised among healthcare professionals regarding the tests’ clinical utility and analytical validity.^8^^,^^9^ The US Food and Drug Administration (FDA) categorized DTC-GTs as medical device, and issued warning letters in 2013 to private companies specifying the negative consequences of the false positive or false negative results for high-risk indications.^10^^,^^11^ The DTC-GTs originating in the USA usually do not obtain country-specific approval in non-US jurisdictions.^12^ Consequently, the worldwide online access consumers have challenged the non-US authorities in enforcing local regulations of internet-based products.^13^ Across the European Member States, a fragmented legislation is present, with different legal frameworks.^14^^,^^15^ The European Society of Human Genetics published a policy document about DTC-GTs, covering recommendations on clinical utility, laboratory quality standards, pre- and post-test counseling and data privacy.^16^ However, individuals who perform DTC-GT may seek additional counseling within the traditional healthcare system, and increase the demand for further unnecessary tests and medical procedures.^17^ A systematic review, published in 2015, that analyzed DTC-GTs in a comprehensive manner, reported an overall low level of knowledge and a high level of interest in purchasing DTC-GTs.^18^ This systematic review included only six European studies^19–24^ conducted in UK,^19–21^ Greece,^24^ Switzerland^22^ and the Netherlands.^23^ Considering the increasing availability of the DTC-GTs and the fragmented regulation in EU countries,^15^ European initiatives are developed aiming to improve genomic literacy among citizens.^25^ Therefore, it is crucial to update the current level of understanding about the European citizens’ awareness on DTC-GTs in order to further contribute to the development of educational strategies for general public. In our study, we summarized the current knowledge, attitude and behavior of European citizens on DTC-GT, by updating the most recently published systematic review.

## Methods

### Search strategy

Our systematic review updates the existing literature synthesis, published by Covolo et al. which covered the period up to October 2014.^18^ We searched PubMed, ISI Web of Science, Embase and Google Scholar databases to retrieve studies published from 1 October 2014 to 30 April 2019. We additionally expanded the search in Scopus database without publication date restriction, since it was not addressed in the previous review. The following query was used in PubMed:

‘direct-to-consumer’ AND (genetic OR genomic) AND (citizen OR citizens OR consumer OR consumers OR participant OR participants OR public OR adopter OR adopters OR population OR populations OR user OR users) AND (knowledge OR attitude OR attitudes OR perspective OR perspectives OR behavior OR behaviors OR opinion OR opinions OR perception OR perceptions OR awareness OR experience OR experiences).

We performed our systematic review according to the Preferred Reporting Items for Systematic Reviews and Meta-Analyses (PRISMA) guidelines^26^ (Supplementary Prisma checklist).

### Inclusion criteria

We included studies in English language, conducted in European countries that reported original data on citizens’ knowledge, attitudes and behaviors toward DTC-GT. In terms of knowledge, we were interested whether the citizens have ever heard about DTC-GTs in general and were aware of their existence. Regarding behavior, we referred to the previous personal use or experience with DTC-GTs, whereas attitudes refer to the motivation for undergoing and/or for refusing the DTC-GTs in the future. Editorials, comments, conference papers and narrative reviews were excluded.

### Selection criteria and data extraction

The first screening was performed via title and abstract by two independent researchers (J.S.; I.H.). In the second step, studies with available full text were carefully reviewed. The reference lists of the included studies were hand-searched for additional relevant publications. Two investigators (J.S.; I.H.) independently extracted data on first author, publication year, study design and setting, data collection period, data collection method, number of participants contacted, final number of participants, response rate, participants’ characteristics and main findings. The required data were reported into evidence tables and the information was double-checked for completeness and accuracy. Discrepancies were resolved through discussions with a third author (SB), until consensus was reached.

### Quality assessment

We assessed the quality of the included studies by using the checklist developed by Kmet et al.^27^ that appraises the quality of primary research papers by using two separate scoring system for quantitative and qualitative studies. This validated tool evaluates the research design, sampling strategy, data collection methods, data analysis, study results and conclusions. When evaluating if the specific criteria were met, 14 items for quantitative studies and 10 for qualitative studies were scored ‘yes = 2, partial = 1, no = 0 or not applicable = N/A’. For each study, the total score obtained across the items was calculated and additionally, the summary score was calculated by dividing the total score by the total possible score. The summary scores are used to define a minimum threshold for inclusion of eligible studies in the systematic review. The cut point selected for article inclusion might be either relatively conservative (e.g. 75%) or relatively liberal (e.g. 55%), for both quantitative and qualitative studies.^27^ Two investigators (J.S.; I.H.) independently scored the specific items of the checklist in each article.

## Results

### Bibliographical search

**Figure 1:**
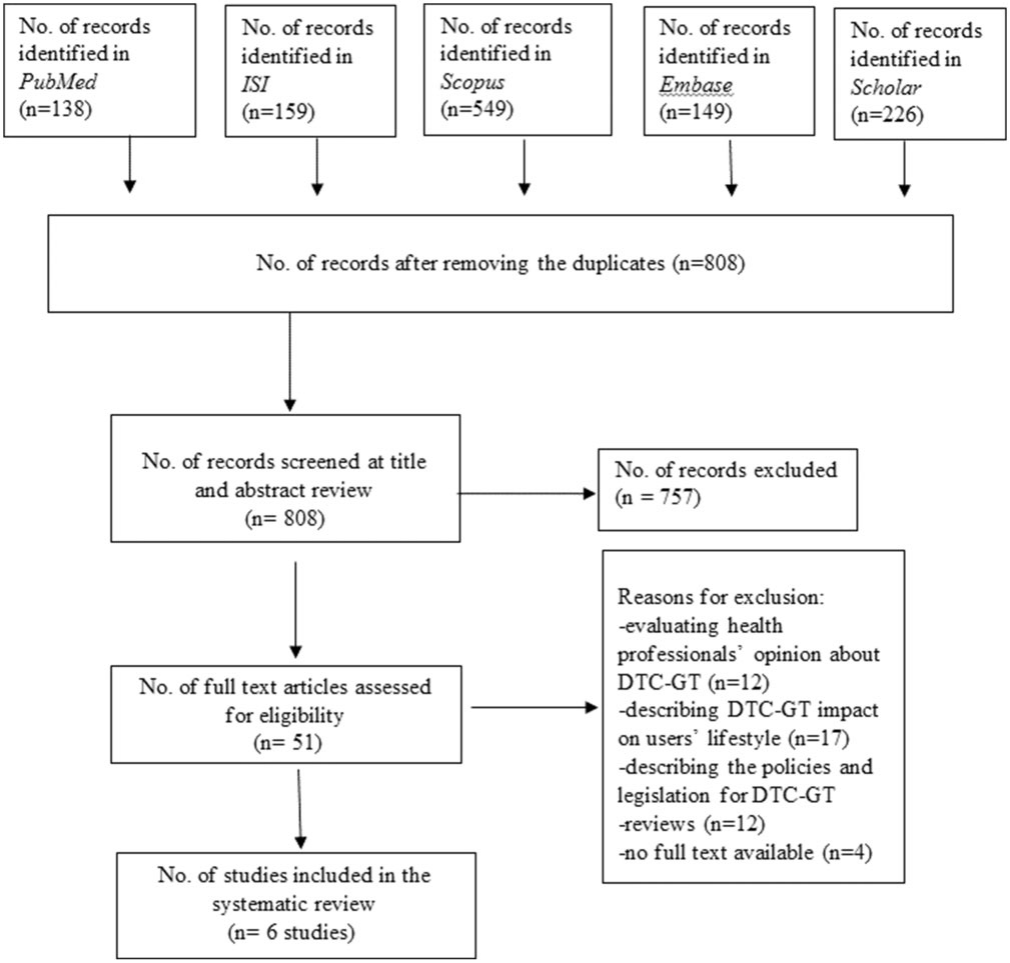
Flowchart of the literature searching process

The search strategy identified a total of 1221 articles in the screening phase from all databases. After removing the duplicates, 808 articles were evaluated via title and abstract screening, of which 51 full-text articles were critically reviewed. We excluded studies that evaluated health professionals’ opinion, reported the country’s legislation and policies and described the DTC-GTs impact on users’ lifestyle. Overall, six articles^28–33^ satisfied the inclusion criteria for this systematic review. No additional studies were included after manually checking the references of the eligible studies. The study selection process is reported in detail in figure 1.

### Study characteristics

Table 1 provides an overview of the main characteristics of the six included studies that covered the period 2015–2018. Two studies were conducted in Italy,^28^^,^^29^ whereas the other four in Switzerland,^30^ Greece,^31^ the Netherlands^32^ and Germany.^33^ One study^33^ applied descriptive methodology by using focus group discussions while five studies^28–32^ implemented quantitative study design (survey). Forty-three German laypeople participated in the qualitative study,^33^ and the majority was highly educated (51% with an academic degree) and female (61%). Among the quantitative studies, the sample size ranged from 145^29^ to 836^32^ subjects. One study was conducted on general population in the Netherlands,^32^ one study on older adults in Switzerland^30^ and three studies on students in Italy^28^^,^^29^ and Greece.^31^

**Table 1:** Characteristics of the six studies included in the systematic review

Study, year	Country	Population and setting	Number of participants contacted	Response rate	Final number of participants	Data collection period	Characteristics of participants
Schaper et al., 2018^33^	Germany	German laypeople from Göttingen, Berlin, Frankfurt, Cologne	NR	NR	43 (7 focus groups)	June 2016–November 2016	Age:18–25 (21%), 26–35 (32%), 36–50 (12%) 51–70 (26%), 70+ (9%)
							Sex: female (61%), male (39%)
							Education: 9 years (5%), 10 years (9%); high school (26%); vocational school (9%); academic degree (51%)
							Average time spent online per day in hours:
							0–1 (16%), 1–2 (37%), 2–4 (26%), 4–6 (7%)
							6+ (12%), none (2%)
Giraldi et al., 2016^28^	Italy	Students enrolled in the Faculty of Medicine at the Università Cattolica del Sacro Cuore, Rome	380	0.471	179	2014	Age: median = 21 years
							Sex: female (59.8%), male (39.7%)
							Academic year: I (45.3%), II (17.9%), III (17.9%), IV (11.7%), V (6.7%)
Mählmann et al., 2016^30^	Switzerland	Older adults attending Seniors’ University, Zurich	800	0.19	151	November 2013–March 2014	Age: mean = 76 years (SD = 6.05)
							Sex: female (45.7%), male (54.3%)
							Education: secondary school (4.6%); vocational education (44.4%); high school (18.5%); university degree (32.5%)
							Internet use: yes (92.1%), no (7.9%)
							Disease in family: yes (31.8%), no (65.65%)
Oliveri et al., 2016^29^	Italy	Subjects with at least bachelor degree from the University of Milan, Milan	250	0.58	145	September 2015–January 2016	Age: mean = 31.41 (SD = 7.58)
							Sex: female (77.9%), male (22.1%)
							Education: 49% had a bachelor’s degree, 51% ranged from master degree to PhD and/or specialization
Stewart et al., 2018^32^	The Netherlands	Online panel members, representative of the Dutch adult	1693	63%	836	June 2017	Age: 18–39 (29.9%), 40–59 (35.8%), 60+ (34.3%)
							Sex: male (50.5%), female (49.5%)
							Education level: low (32.5%); middle (43.3%), high (24.2%)
Mavroidopoulou et al., 2015^31^	Greece	Undergraduate, postgraduate, doctoral students from various disciplines and university sites	NR	NR	725	January 2014–July 2014	Age: 71.9% was ≤25, 20.1% was 26–30, 85% were >30 years
							Sex: female (68.1%), male (31.9%)
							Education level: undergraduate (81.4%); postgraduate (14.2%); doctoral (4.4%)
							Study field: healthcare/biomedical (45.3%); non-biomedical sciences (54.7%)

In the Netherlands, 836 participants were representative of the Dutch adults, with an average education level and with a slight male predominance (50.5%). Among the studies conducted on students, most of the participants were female (59.8% in Rome,^28^ 77.9% in Milan^29^ and 68.1% in Greece^31^) The study in Rome^28^ included undergraduate students of medicine (median age = 21), while the Milan study^29^ included both bachelor degree and advanced degree students, from various disciplines (mean age= 31.41 ± 7.58). The Greek study^31^ included undergraduate students, (81.4%) with the majority less than 25 years of age (71.9%). Swiss older adults (*N* = 151; mean age = 76 years ± 6.05) were mostly male (54.3%) and had an above-average educational level (32.5% university degree, 18.5% high school and 44.4% vocational education).^30^

### Quality assessment

The quantitative studies were estimated with quality assessment summary score ranging from 94% to 100% (Supplementary table S1). Both reviewers assigned the same overall quality score: 100% in four studies^28^^,^^29^^,^^31^^,^^32^ and 94% in one study.^30^ In the qualitative study^33^ both reviewers assigned the same overall score of 75% (Supplementary table S2), evaluating the study sample size as insufficient with respect to the outcome. In conclusion, all the quantitative and qualitative studies reached the defined conservative threshold for inclusion (75%) in the systematic review. Overall, the inter-rater agreement by item and the inter-rater agreement for overall scores were 100% among the two researchers.

### Main findings

Considering the citizens’ knowledge and awareness toward DTC-GTs, most of German laypeople were unaware of its existence, whereas, one-third of Swiss older adults and 28.5% of adults in the Netherlands were aware of DTC-GTs for disease-related purposes. Medical students in Rome had the highest level of awareness (45.3%), followed by Milan and Greek students, 33% and 30.1%, respectively. In Greece, postgraduate students from biomedical disciplines were more likely to be aware of DTC-GT. As for the citizens’ behaviors, 0.2% of adults in the Netherlands and only one participant among Swiss older adults had pursued a DTC-GT. Subjects with DTC-GT experience were present in each of the discussion groups of German laypeople. Students in Rome and Milan were without previous personal experience, whereas 3.4% of Greek students had pursued a DTC-GT. Regarding the attitudes, overall, participants expressed interest in undergoing a DTC-GT, with differences according to country and population group. A total of 56% of Swiss older adults and 12.6% of Dutch adults would probably consider undergoing DTC-GT in the future. German laypeople expressed preference to DTC-GTs’ provision within the traditional public healthcare system, if there were enough quality control. Among the students, 73% of students in Rome were interested in purchasing a DTC-GT. A total of 76% of Greek students were prone to consult a doctor before the test and 51.6% would order the test if it was for free.

Knowing the personal risk of a certain disease, probably cancer or CVD was the main reason for undergoing a DTC-GT in over half of the participants: 86% of students in Rome, 56% of Greek students, 21.4% of students in Milan and 70.2% of Swiss older adults. More than 60% of students in Greece and Rome were interested in knowing the risk of passing disease predisposition onto the children. Swiss older citizens stated the same reason (53.6%), along with the willingness to contribute their genetic data to scientific research (63.1%). Learning about the current health status, knowing the sensitivity to certain medication and obtaining information about the genetic ancestry were additional reasons for pursuing a DTC-GT. On the other hand, participants that were not interested in purchasing DTC-GTs claimed a lot of scientific and ethical concerns. Italian students and Swiss older adults raised the concerns that the test results might cause worry and affect their behavior. Concerns about data privacy were common between Greek students (47.8%), although very low among the students in Rome (19.1%). German laypeople were critical regarding the reliability of the commercial companies whereas Italian (24.2%) and Greek (41.8%) students expressed their skepticism toward the reliability of the test results. Summarized findings with regard to citizens’ knowledge, behavior and attitudes are reported in table 2.

**Table 2:** Main findings from the six studies included in the systematic review

Study	Year	Population	Data collection method	Main findings
Schaper et al.^33^	2018	43 German laypeople	Qualitative approach with seven focus groups, asking for: attitudes, perceptions and assumed ethical implications toward DTC-GT	Knowledge: participants were unfamiliar with DTC-GTBehavior: in each focus group, a participant had performed DTC-GTAttitudes: critical toward health-related and predictive DTC-GT supportive of lifestyle DTC-GT reserved regarding commercial provision of DTC-GT opposed the legal restrictions on commercial distribution of GT
Giraldi et al.^28^	2016	179 medical students from Italy	A self-administered anonymous questionnaire asking for: demographic characteristics awareness and experience of DTC-GT (dichotomous yes/no questions); interest in undergoing or not DTC-GT (multiple-choice questions) the willingness to make the data available for research (dichotomous yes/no questions) the willingness to know the results of the test (dichotomous yes/no questions) the institutions they would support participating in a genetic study via DTC-GT (multiple-choice questions) opinion on DTC-GT after filling out the questionnaire	Knowledge:45.3% were aware of DTC-GTBehavior: none of the respondents has ever performed a DTC-GTAttitudes:73% would undergo DTC-GT, of which 88% would make data available for research and 92% would like to know the test resultsReasons for the interest in undergoing DTC-GT: knowing the risk of certain diseases (86.4%) knowing the risk of passing disease predisposition onto the children (60.6%) knowing the personal characteristics (52.3%) knowing the sensitivity to certain medication (49.2%) contributing genetic data to scientific research (47.7%)27% refrain from undergoing DTC-GT (*n* = 47)
				Reasons for not undergoing DTC-GT: concerns about the test validity (48.9%) concerns that the results might cause worry (42.6%) disinterest (27.7%) concerns about the data privacy (19.1%)
Mählmann et al.^30^	2016	151 Swiss older adults	An anonymized voluntary self-completion survey with 31 multiple-choice and 7-point Likert scale questions, asking for: demographic variables awareness about personal genomics services, motivation for testing and concerns about genomic results attitudes toward research participation in genomic research studies attitudes toward sharing genomic data in genomic research studies	Knowledge:One-third of the respondents were aware of DTC-GT
				Behavior: 0.6% had previously performed a DTC-GTAttitudes: 56% were interested in undergoing DTC-GT
				Reasons for the interest in undergoing DTC-GT: knowing the risk of certain disease (70.2%) contributing the data to scientific research (63.1%) knowing the sensitivity to certain medication (57.1%) knowing the genetic ancestry (54.8%) knowing the risk of disease predisposition onto the children (53.6%) knowing personal genetic traits (42.9%)44% participants were not interested in undergoing DTC-GT
				Reasons for not undergoing DTC-GT: concerns that the results might cause worry (45.5%) concerns about the test validity (42.4%) disinterest/lack of utility (42.4%) concerns about the data privacy (27.3%)
Oliveri et al.^29^	2016	145 subjects with at least bachelor degree from Italy	Online survey asking for: demographic variables health orientation scale knowledge and attitudes toward DTC-GT level of perceived knowledge about genetic risk, genetic testing and DTC-GT (Likert scale or yes/no questions) sources of information (open question) motivation for accepting or refusing DTC-GT, possible impact on current and/or future health behaviors and decisions (closed questions) intentions to undergo the DTC-GT (motivation levels on an 0–10 scale)	Knowledge: 33% were aware of DTC-GTBehavior: Not reported
				Attitudes:Reasons for the interest in undergoing DTC-GT: knowing current health status (14.4%) adopting health behaviors (15.2%) increasing early detection of the disease (21.4%)
				Reasons for not undergoing DTC-GT: concerns about the reliability of the results (24.2%) concerns that the results might cause worry and impact future behaviors (17.2%)
Stewart et al.^32^	2018	836 online panel members, representative of the Dutch adult, based on age, gender and education level.	Online survey, sent by mail, asking for: demographic variables (including a brief introduction on DTC-GT) awareness of DTC-GT for disease-related purposes (dichotomous yes/no questions) previous use of DTC-GT for disease-related purposes acceptability of the DTC-GT (Likert 5-point scale, 1—completely unacceptable to 5—completely acceptable) consideration to undergo DTC-GT for disease-related purposes at some time in the future (5-point scale: 1—certainly no to 5—certainly yes) intention to undergo DTC-GT for disease-related purposes in the next year (5-point scale :1—certainly no to 5—certainly yes)	Knowledge: 28.5% were aware of DTC-GT for disease-related purposes Behavior: 0.2% had previously performed a DTC-GT for disease-related purposesAttitudes: 12.6% considered undergoing DTC-GT for disease relatedpurposes in the distant future 5.5% intended to undergo DTC-GT for disease-related purposes in the coming year
Mavroidopoulou et al.^31^	2015	725 undergraduate postgraduate and doctoral students from Greece	Printed and online survey asking for: demographic variables (including a brief introduction on DTC-GT) 24 closed-ended questions (Likert, dichotomous and buying propensity questions): 11 questions on awareness, interest and reasons to take/refuse DTC-GT 13 questions after introducing hypothetical scenario to assess understanding of the DTC-GT results and their impact on psychology and future actions	Knowledge: 30.1% were aware of DTC-GT (39% healthcare/biomedical sciences students; 24% non-biomedical)
				Behavior: 3.4% had previously performed a DTC-GT
				Attitudes: 61.3% would agree to take the test after a briefing 54.9% interested in DTC-GT for a serious disease (cancer or CVD) and 76% of them would consult their doctor before 50.2% interested in a DTC-GT for metabolism or genealogy 9.7% would order a DTC-GT if it costs 500€, 51.6% if the test is free
				Reasons for the interest in undergoing DTC-GT: learning about their health (>60%) warning their children (>60%) their doctor can monitor their health (>60%) changing lifestyle (>60%)
				Reasons for not undergoing DTC-GT: knowing the risk of certain disease (55.7%) concerns about the data privacy (47.8%) concerns about the reliability of the results (46.9%) concerns about the utility of the test (27.7%)

DTC-GT, direct-to-consumer genetic testing; CVD, cardiovascular disease.

## Discussion

Our updated systematic review on European citizens’ perspectives toward DTC-GTs included six studies published from October 2014 to April 2019, which were conducted in Italy,^28^^,^^29^ Switzerland,^30^ Greece,^31^ the Netherlands^32^ and Germany.^33^ Overall, European citizens had quite a low level of awareness and a high level of interest in purchasing DTC-GTs, with differences by country and population group. The most common reason for undergoing a DTC-GT was the willingness to know the risk predisposition for a certain serious disease. The main reason of refraining from undergoing a DTC-GT was the worry that results might cause unnecessary distress and anxiety. The low level of knowledge and awareness as well as high level of interest are in line with the findings of the systematic review by Covolo et al.^18^ However, the main reasons for interest in DTC-GT reported in the previous review were monitoring health status and curiosity.

A high level of awareness among students, particularly of biomedical sciences, is *per se* expected in highly educated subjects,^34^ as reported in a previous study, with 65.7% of Swiss students being aware of DTC-GT, most of whom were enrolled in natural sciences.^22^ A moderate level of awareness was found even between Swiss older citizens,^30^ which was probably related to the media attention during the revision of the laws on GTs.^22^ Among the general population, a low level of awareness (28.5%) was reported from a 2018 survey in the Netherlands^32^ and even lower rates (13%) have been described earlier in the UK.^19^

Considering the citizens ’attitudes, the fact that German laypeople would purchase the test if it was prescribed by their doctor^33^ may oppose the legislative framework currently in force in Germany, highlighting that the legal restrictions on commercial distribution of GTs were strongly opposed. Notably, the interest in purchasing DTC-GTs differed among the Dutch general population across the years. In 2018, up to 12.6% of citizens would consider undergoing a DTC-GT for health-related purposes^32^ whereas, in 2010, half of the respondents expressed interest in DTC-GTs for a genetic predisposition to specific diseases. In both studies, the lower-educated respondents were more interested. Low interest (17.9%) was observed even among general population in Greece, while 82% of the participants would consult a physician.^24^ In some countries, the interest in DTC-GTs was cost dependent. In Greece, for instance, 54.8% of the general public would purchase the test even though the cost would not be reimbursed^24^ whereas, 50.6% of students would do the same only if free of charge.^31^ Similarly, test pricing was a decisive factor in the decision to undertake the test in the UK.^19^ The emerging culture of the consumer empowerment may lead to a higher interest in purchasing DTC-GTs, especially considering the rapid technological developments and decreasing costs of genomic testing nowadays.^35^ The willingness to provide genetic data to scientific research, stated by Swiss students^22^ and older adults^30^ and Italian medical students,^28^ has been described as a moral duty to contribute to the society’s common good and to the medical advancements for the sake of next generations.^36^ The participants’ concerns regarding the tests’ clinical utility and analytical validity have been extensively discussed in the literature,^8^^,^^9^ as well as data privacy and lack of results’ confidentiality.^37^^,^^38^ The concerns about data privacy are of particular importance considering that genomic re-identification strategies can easily determine people’s surnames by using publicly available sequencing data as well as metadata of anonymous participants.^39^

However, the very small number of actual users of DTC-GTs among the EU countries does not allow us to predict how the general public would understand the tests’ results. A recent meta-analysis, on behavioral changes after performing DTC-GTs, showed that anxiety and worry among the users were rather low and the actual effects of DTC-GT on behavior changes are modest, especially when results are delivered without additional lifestyle counseling.^40^ This is in contrast with the studies included in our updated review, where Italian and Greek students were eager to perform DTC-GT in order to learn about the current health status and subsequently change lifestyle behaviors. This controversy might be explained with the fact that young individuals, especially biomedical students are more health conscious and have a strong belief on the benefits of the GTs.

Some limitations should be taken into consideration when interpreting the results of our updated systematic review. We considered only available published studies, leading to a possible publication bias. Moreover, we included only English language studies. It should be noted that the eligible studies were not representative of the general population, since they included highly educated individuals, small sample sizes and low response rates. Despite the small sample sizes, individual studies were methodologically sound and may be considered as the initial step for future large-scale research. Further research activities should use representative sample of the general populations and should be focused on the expectations and behavioral changes among European users. Nevertheless, our study adds up to the previous research and provides an up-to-date understanding of the population-level awareness on DTC-GT among the European countries.

Overall, a limited number of studies, precisely 12, have been focused on European citizens’ perspectives toward DTC-GTs. In contrast to European scenario, 37 studies conducted in USA were included in the systematic review published in 2014 by Covolo et al. In the last 5 years, a considerable amount of US studies have been carried out (41–47 in Supplementary data), mostly related to differences in knowledge and attitudes in socio-demographic groups. A national survey reported that awareness in USA toward DTC-GTs increased from 31% to 38% between 2007 and 2014 (42 in Supplementary data). Moreover, a recent study that analyzed the data of 17 403 respondents revealed that the awareness increased from 29.23% in 2007 to 56.78% in 2017 (44 in Supplementary data). This increase in awareness might be linked to the legal framework that was implemented in the USA. FDA has been granting market approval for certain types of DTC-GTs, opening a way for consumer companies to implement strong advertising campaigns and increase access of individuals to these tests (48 in Supplementary data). The enormous advancements in the genetic field have provided a growing application of GTs for diagnostic, predictive and treatment purposes in healthcare. In terms of the high availability (49 in Supplementary data), increased demand and the limited number of studies conducted in European countries, DTC-GTs should be considered as an emerging public health issue. Uninformed consumers might be undergoing unnecessary tests which might lead to overconsumption of health services without adding any value (50 in Supplementary data). Consumers’ ability to understand the results and to seek follow-up genetic counseling might be related to their level of knowledge (51 in Supplementary data). Therefore, our findings highlight the importance of tracking the citizens’ perceptions and misperceptions, in order to develop recommendations related to their educational needs. Educational and counseling strategies should be provided on the national levels aiming to increase the general publics’ understanding of genetic information in order to make appropriate health decisions.

## Conclusion

European citizens expressed low level of awareness towards DTC-GT, which differed by country and group population. The majority showed a high interest in purchasing a DTC-GT, having as the main reason the willingness to know the risk predisposition to a common disease. The citizens that were not interested in purchasing a DTC-GT raised concerns about tests' clinical and analytical validity, clinical utility, data privacy and results' confidentiality.

## Supplementary data

Supplementary data are available at *EURPUB* online.

## Supplementary Material

ckz246_Supplementary_Data
