# Supplementary material for: European citizens’ perspectives on direct-to-consumer genetic testing: an updated systematic review
Source: Eur J Public Health. 2020 May 3;33(5):947–53. doi: 10.1093/eurpub/ckz246 (PMC11227739; doi:10.1093/eurpub/ckz246)
Supplement: ckz246_Supplementary_Data [file ckz246_supplementary_data.zip › ckz246_Supplementary_Data/ejph-2019-07-srm-0608-File006.docx]

**Supplementary Table 1.** Quality assessment of quantitative studies included in the systematic review

| Study  Year | Giraldi 2016 | Mählmann 2016 | Oliveri 2016 | Stewart 2018 | Mavroidopoulou 2015 |
| --- | --- | --- | --- | --- | --- |
| *Objective* | 2 | 2 | 2 | 2 | 2 |
| *Study design* | 2 | 2 | 2 | 2 | 2 |
| *Method of subject selection* | 2 | 2 | 2 | 2 | 2 |
| *Subject characteristics* | 2 | 2 | 2 | 2 | 2 |
| *Random allocation (if applicable)* | N/A | N/A | N/A | N/A | N/A |
| *Blinding of investigators (if applicable)* | N/A | N/A | N/A | N/A | N/A |
| *Blinding of subjects (if applicable)* | N/A | N/A | N/A | N/A | N/A |
| *Outcome and exposure definition/means of assessment of bias* | 2 | 1 | 2 | 2 | 2 |
| *Sample size (if applicable)* | N/A | N/A | N/A | N/A | N/A |
| *Analytic method described* | 2 | 2 | 2 | 2 | 2 |
| *Estimate of variance (if applicable)* | N/A | N/A | 2 | 2 | N/A |
| *Controlled for confounding (if applicable)* | N/A | N/A | N/A | N/A | N/A |
| *Results detailed* | 2 | 2 | 2 | 2 | 2 |
| *Conclusions* | 2 | 2 | 2 | 2 | 2 |
| *Total sum* | 16 | 15 | 18 | 18 | 16 |
| *Total possible sum* | 16 | 16 | 18 | 18 | 16 |
| *Total score (%)* | 100 | 94 | 100 | 100 | 100 |
