# Supplementary material for: European citizens’ perspectives on direct-to-consumer genetic testing: an updated systematic review
Source: Eur J Public Health. 2020 May 3;33(5):947–53. doi: 10.1093/eurpub/ckz246 (PMC11227739; doi:10.1093/eurpub/ckz246)
Supplement: ckz246_Supplementary_Data [file ckz246_supplementary_data.zip › ckz246_Supplementary_Data/ejph-2019-07-srm-0608-File007.docx]

**Supplementary Table 2**. Quality assessment of qualitative studies included in the systematic review

| Study Year | Schaper  2018 |
| --- | --- |
| *Objective* | 2 |
| *Study design* | 2 |
| *Context for the study* | 2 |
| *Connection to a theoretical framework* | 0 |
| *Sampling strategy* | 1 |
| *Data collection* | 2 |
| *Data analysis* | 2 |
| *Verification procedure* | 0 |
| *Conclusions* | 2 |
| *Reflexivity of the account* | 2 |
| *Total sum* | 16 |
| *Total possible sum* | 20 |
| *Total score (%)* | 70 |
