# Supplementary material for: European citizens’ perspectives on direct-to-consumer genetic testing: an updated systematic review
Source: Eur J Public Health. 2020 May 3;33(5):947–53. doi: 10.1093/eurpub/ckz246 (PMC11227739; doi:10.1093/eurpub/ckz246)
Supplement: ckz246_Supplementary_Data [file ckz246_supplementary_data.zip › ckz246_Supplementary_Data/ejph-2019-07-srm-0608-File008.docx]

**References**

41. Gollust SE, Gray SW, Carere DA, Koenig BA, Lehmann LS, McGuire AMYL, et al. Consumer Perspectives on Access to Direct-to-Consumer Genetic Testing: Role of Demographic Factors and the Testing Experience. Milbank Q. 2017 Jun 1;95(2):291–318.

42. Apathy NC, Menser T, Keeran LM, Ford EW, Harle CA, Huerta TR. Trends and Gaps in Awareness of Direct-to-Consumer Genetic Tests From 2007 to 2014. Am J Prev Med. 2018 Jun 1;54(6):806–13.

43. Salloum RG, George TJ, Silver N, Markham MJ, Hall JM, Guo Y, et al. Rural-urban and racial-ethnic differences in awareness of direct-to-consumer genetic testing. BMC Public Health. 2018 Feb 23;18(1).

44. Wei H, Chen Z, Cobran E. Perceived Cancer Risk and Evaluation of Public Awareness of Direct-to-Consumer Genetic Tests in the United States. Value Heal. 2018;21:S127.

45. Agurs-Collins T, Ferrer R, Ottenbacher A, Waters EA, O’Connell ME, Hamilton JG. Public Awareness of Direct-to-Consumer Genetic Tests: Findings from the 2013 U.S. Health Information National Trends Survey. J Cancer Educ. 2015 Dec 1;30(4):799–807.

46. Nicanor Austriaco. Direct-to-consumer genetic testing in the college classroom: Knowledge, attitudes, and concerns of introductory biology students. PeerJ. 2014;1(2):1–47.

47. Baptista NM, Christensen KD, Carere DA, Broadley SA, Roberts JS, Green RC. Adopting genetics: Motivations and outcomes of personal genomic testing in adult adoptees. Genet Med. 2016 Sep 1;18(9):924–32.

48. FDA allows marketing of first direct-to-consumer tests that provide genetic risk information for certain conditions Share Tweet Linkedin Email Print. 2017; Available from: https://www.fda.gov/news-events/press-announcements/fda-allows-marketing-first-direct-consumer-tests-provide-genetic-risk-information-certain-conditions

49. de Paor A, Blanck P. Precision Medicine and Advancing Genetic Technologies—Disability and Human Rights Perspectives. Laws. 2016;5(3):36.

50. Ramos E, Weissman SM. The dawn of consumer-directed testing. Am J Med Genet PART C-SEMINARS Med Genet. 2018 Mar;178(1, SI):89–97.

51. Leighton JW, Valverde K, Bernhardt BA. The general public’s understanding and perception of direct-to-consumer genetic test results. Public Health Genomics. 2011;15(1):11–21.
